# From Astrophysics to Perovskites: Why Saha Physics Cannot Describe the Exciton to Free Electron–Hole Pairs Statistics in Semiconductors

**DOI:** 10.1021/acsaom.6c00127

**Published:** 2026-06-04

**Authors:** Giuseppe Ammirati, Faustino Martelli, Daniele Catone, Patrick O’Keeffe, Stefano Turchini

**Affiliations:** 1 EuroFEL Support Laboratory (EFSL), 204549 Istituto di Struttura della Materia - CNR (ISM-CNR), Via del Fosso del Cavaliere 100, Rome 00133, Italy; 2 EuroFEL Support Laboratory (EFSL), Istituto di Struttura della Materia - CNR (ISM-CNR), Monterotondo Scalo 00015, Italy

**Keywords:** semiconductors, excitons, Mott transition, Saha equation, halide perovskites, electronic properties

## Abstract

The Saha equation has long served as the standard model for describing thermodynamic equilibrium between neutral atoms and their ionized counterparts in astrophysical plasmas. Over the past decade, this formalism has been increasingly adopted to describe the equilibrium between excitonic and free electron–hole (e–h) populations in semiconductors, especially halide perovskites. Here, we argue that this application is fundamentally limited. The Saha equation has been developed for systems in thermodynamic equilibrium, while a photoexcited semiconductor is out of equilibrium. Moreover, in all semiconductors, strong many-body interactions, density-dependent Coulomb screening, and band gap renormalization invalidate the key Saha assumption of a fixed exciton binding energy. As a result, Saha-based phase diagrams fail to capture the Mott transition, the density-driven transition from an excitonic gas to a plasma of free electron–hole pairs, and to describe semiconductor laser operation. We hope that our considerations help the perovskite community correctly describe the electronic population in their works.

## Graphical abstract

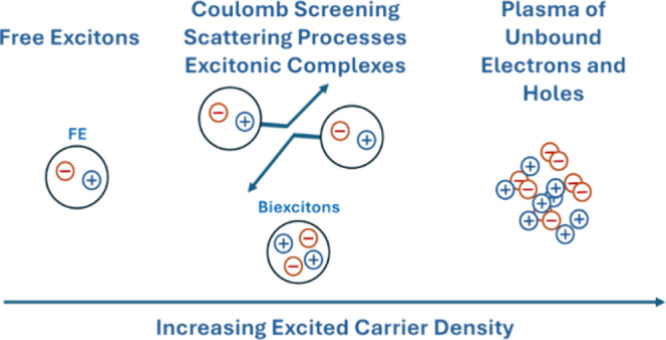

## 1 Introduction

Photoexcitation in semiconductors generates electron–hole (e–h) pairs whose character, stability, and collective behavior critically depend on carrier density and temperature. Depending on excitation conditions, these photoexcited carriers may exist as free electrons and holes, form excitons via Coulomb interaction, or participate in more complex many-body configurations. Understanding the balance between excitons and unbound e–h states is therefore central to the interpretation of optical and transport experiments and to the modeling of optoelectronic devices.

Although this physics has been well established for decades, in recent years, this problem has gained renewed attention in the context of hybrid halide perovskites. To rationalize the relative populations of excitons and free carriers, several works have adopted models based on the Saha equation, originally developed to describe ionization equilibrium in astrophysical plasmas. Within this framework, excitons are treated as hydrogen-like neutral species, while free electrons and holes are regarded as an ionized plasma, with their relative populations determined by temperature and a fixed exciton binding energy.

While this analogy is appealing in its simplicity, its applicability to semiconductors is highly nontrivial. The Saha equation relies on assumptions of thermodynamic equilibrium, weak interactions, and fixed energy levels. In contrast, photoexcited semiconductors are intrinsically in nonequilibrium and constitute many-body systems, in which Coulomb screening, band-structure renormalization, Pauli exclusion, and nonideal plasma effects play a dominant role. As a result, the exciton binding energy is not a fixed material constant but a density-dependent quantity.

In this work, we show that simplified Saha models, widely used in the perovskite literature, lead to unphysical predictions that contradict both established semiconductor theory and experimental evidence.

## 2 Photoexcitation In Semiconductor Physics

Photoexcitation in semiconductors gives rise to electron–hole pairs whose character and stability critically depend on carrier density and temperature, constituting a well-established physical framework in semiconductor physics. While temperature controls the thermal occupation of electronic states and the kinetic energy of carriers, the nature of the photoexcited electron–hole pairs is primarily governed by carrier density through Coulomb and many-body interactions. At low temperatures and low excitation densities, electrons and holes behave as well-defined quasiparticles and will bind into free excitons (FE) with hydrogenic character. In this dilute regime and with thermal energy weaker than the Coulomb one, excitons can be regarded as weakly interacting composite bosons, coexisting with a small population of free carriers and, in some materials, defect-bound complexes. As the photoexcited carrier density increases, interactions between these species become increasingly important. Coulomb interactions give rise to scattering processes and higher-order bound states such as biexcitons and trions, while phase-space filling and exchange interactions modify the single-particle spectrum. In this intermediate-density regime, both the photoexcited carrier density and temperature influence the relative stability of excitonic and free-carrier populations; however, the ideal gas limit is not valid for that system.^1^

At sufficiently high carrier densities, exciton wave functions begin to overlap, and many-body effects dominate the interactions. Coulomb screening reduces the effective electron–hole attraction, while band gap renormalization lowers the continuum edge. Simultaneously, Pauli exclusion restricts the available phase space for excitonic bound states. The sum of these effects leads to a progressive reduction, and eventual collapse, of the exciton binding energy at a critical carrier density known as the *Mott density*.^1^ It is important to underline that those processes are not driven by thermal dissociation but by interaction-induced renormalization. Beyond this point, excitons lose their identity as stable quasiparticles, and the system crosses over into a collective, *degenerate electron–hole plasma* (EHP). Eventually, below a critical temperature and in the presence of long carrier lifetime, many-body renormalization of the electronic eigenstates in the electron–hole plasma (EHP) will lead to the formation of an electron–hole liquid (EHL).^2,3^ Obviously, the model also holds at lower dimensions.^4^

This framework, established experimentally through the pioneering work of Thomas in Ge^3^ and Shah in Si^5^ (see Figure 1a for the reported FE-EHP–EHL phase diagram of Si^5^), has since been extensively validated in semiconductors and provides the natural theoretical framework for describing exciton stability as a function of the photoexcited carrier density.

**Figure 1:**
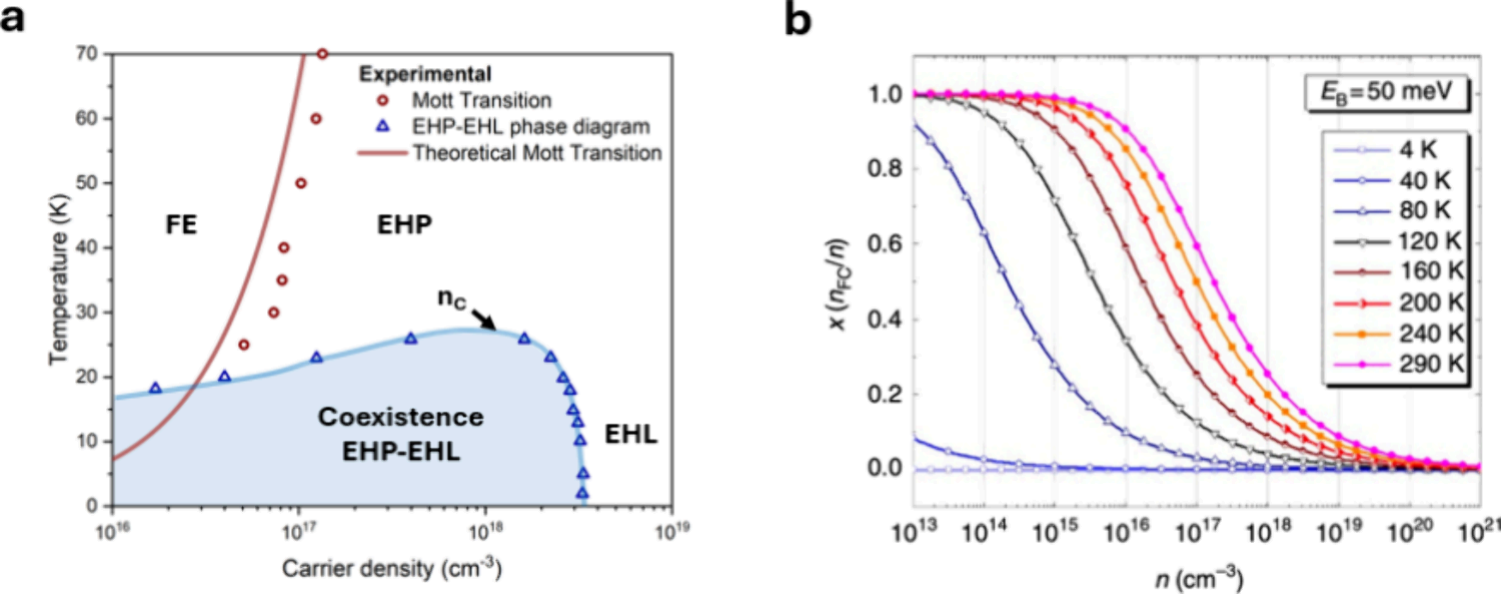
(a) Electronic phase diagram of Si obtained experimentally by Shah et al.^5^ Adapted with permission from ref 5 [Copyright 1977 American Physical Society]. (b) Simulation of the free charge fraction over the total excitation density in the Saha equation regime as reported by D’Innocenzo et al.^10^ Reprinted with permission from ref 10 [Copyright 2014 Springer Nature].

Any model aimed at describing the equilibrium between excitons and free carriers in semiconductors must therefore account explicitly for density-dependent screening, band gap renormalization, and temperature by considering the proper particle statistics.^1^ As we show below, these requirements are fundamentally incompatible with the assumptions underlying the Saha equation.

## 3 The Saha Equation: From Astrophysics to Solid-State Physics

The Saha equation was formulated to describe ionization equilibrium in astrophysical plasmas, where it provided a solution to the problem of interpreting stellar spectra. Astronomical observations had revealed that stars with nearly identical chemical compositions exhibited markedly different absorption lines. Meghnad Saha resolved the problem by showing that temperature, not composition, governs the ionization balance in stellar atmospheres, by relating the degree of atomic ionization to temperature and particle density under conditions of thermodynamic equilibrium. In particular, the Saha equation expresses the balance between neutral atoms and their ionized counterparts as a function of temperature and density, assuming that the system is in detailed balance and can be treated as an ideal gas. These assumptions are valid in dilute stellar atmospheres, and for this reason, the Saha equation has become a standard tool in astrophysics and plasma physics.

However, the validity of the Saha formalism rests on a set of restrictive assumptions that must be kept in mind to properly define its domain of applicability. First, the system must be in thermodynamic equilibrium, such that ionization and recombination processes satisfy a detailed balance. Second, particle interactions must be sufficiently weak so that the plasma can be treated as ideal. Third, the bound-state energies entering the equation are assumed to be fixed and well-defined, independent of particle density.^6,7^

When these conditions are violated, the Saha equation is known to fail. In dense plasmas, strong interactions and pressure ionization lead to the dissolution of bound states even when Saha statistics would predict their stability.^8^

Conversely, in very low-density plasmas, collisions occur too infrequently to establish detailed balance, and ionization dynamics must be described by collisional–radiative models rather than equilibrium thermodynamics. These breakdowns are well documented in the plasma physics literature and highlight that Saha statistics are not universally applicable, even within their original astrophysical context.^9^

Inspired by its conceptual simplicity and the apparent similitude of the two systems, the Saha equation was later adapted to semiconductor physics to describe the balance between bound excitons and free e–h pairs. The analogy is appealing: excitons behave like neutral atoms, while free carriers resemble an ionized plasma. Saha statistics can be hence reformulated for a semiconductor as

$$\frac{x^{2}}{1-x}=\frac{1}{n}{\left(\frac{2\pi \mu k_{B}T}{h^{2}}\right)}^{3/2}e^{-E_{b}/k_{B}T}$$ 1

where *n* is the total density of excitation *n* = *n*_FC_ + *n*_exc_, *x* is the fraction of free charges over the total density of excitation, μ is the reduced mass of the exciton, *h* is the Planck’s constant, *k*_B_ is the Boltzmann constant, *T* is the temperature, and *E*_b_ is the exciton binding energy.^10^ The result of such analysis is shown in Figure 1b derived from ref 10. This paper by D’Innocenzo and co-workers has become a seminal one in the context of hybrid halide perovskites. Although some early warning about the use of the Saha equations without considering many-body effects in that context appeared in the literature,^11^ several hundred papers have adopted its simplified Saha model in which the exciton binding energy is treated as a constant, independent of carrier density. In these approaches, occupation effects, screening, and band gap renormalization are neglected, and the Saha equation is applied in its ideal gas form.^10,12–16^

Surprisingly, the D’Innocenzo paper was inspired by a paper published in 1997 by Cingolani et al., in which the authors implemented a Saha-type statistics explicitly introducing the many-body interactions to preserve physical consistency.^17^ In particular, the exciton binding energy was described as dependent on the carrier density through Coulomb screening, phase-space filling, and bandgap renormalization. When these many-body corrections are included self-consistently, the resulting phase diagrams recover the expected Mott transition behavior: Exciton populations decrease with increasing carrier density, eventually vanishing as the binding energy collapses.

The simplification introduced in ref 10 changes the physical predictions of the model. Because the exciton binding energy is held fixed, increasing carrier density does not induce the Mott transition through screening. Conversely, such models predict that the exciton population can increase with excitation density and even dominate over free carriers at high densities, while free carriers prevail at low excitation, as shown in Figure 1b taken from ref 10. The most striking example showing the inconsistency between Saha statistics and semiconductor physics is the example of the conventional semiconductor lasers.^18,19^ Optical gain in semiconductors requires population inversion, which is achieved when the electron and hole quasi-Fermi levels separate sufficiently for the chemical potential to exceed the renormalized electronic band gap.^20^ This condition implicitly involves the formation of an EHP and requires the occurrence of the Mott transition, with the ensuing formation of an electron–hole plasma. Consequently, excitons cannot serve as the gain medium in conventional semiconductor lasers, and lasing cannot occur in a regime dominated by stable bound excitons. Nevertheless, within the picture of the Saha equation, the semiconductor lasers would operate in an excitonic regime, as suggested, e.g., in ref 21.

The failure of a fixed-binding-energy Saha model is not a quantitative discrepancy but a qualitative one by construction: First, it describes a system in thermodynamic equilibrium, which is not the case of photoexcited semiconductors; second, the fundamental interactions that govern the high-excitation regime are excluded. As a result, it cannot capture the density-driven Mott transition and instead predicts an unphysical scenario. At the same time, it predicts the dominance at low carrier densities of the free-carrier population at any temperature,^10,16,21^ a result which is in stark contrast with what is well established by the semiconductor physics. Indeed, if the thermal energy is lower than the excitonic binding energy, if you excite an electron in the conduction band creating a hole in the valence band, then the Coulomb attraction between the electron and the hole will form one exciton. As the carrier density increases, the Coulomb screening will allow the formation of free electron–hole pairs.

Taken together, these considerations clarify that the use of the simplified Saha statistics is not appropriate to describe the relative population of excitons and free carriers in semiconductors where the Mott model applies. If used, then the applicability of Saha statistics to semiconductors is highly conditional, since they yield predictions that are incompatible with both established semiconductor theory and experimental evidence. The introduction in the model of many-body effects qualitatively results in an agreement with the Mott model, however resulting in a more complicated tool than the Mott criterion.

As mentioned above, despite the early publication of papers that criticizes the use of the Saha statistics in semiconductors,^11^ only recently has the perovskite community begun to critically reassess the widespread use of fixed-binding-energy Saha models. Papers reporting experiments in 2D and 3D perovskites have reported clear contradictions when the Saha predictions are used, driven by dynamic screening and the proximity to the Mott transition.^15,22–26^

## Conclusions

Although the Saha equation provides an elegant description of the ionization equilibrium in dilute, weakly interacting plasmas, its validity is confined to a narrow regime defined by thermodynamic equilibrium, ideal gas behavior, and fixed energy states. Even within its original astrophysical context, these assumptions are known to fail at both low and high densities, requiring more sophisticated treatments.

When transposed to photoexcited semiconductors, which are in nonequilibrium conditions, the limitations of the Saha formalism become fundamental rather than technical. Exciton stability in semiconductors is governed by the carrier density, which induces changes in the dielectric screening, band gap renormalization, Pauli blocking, and degeneracy effects, all of which invalidate the notion of a fixed exciton binding energy.

The classical Saha model inverts the expected density dependence of exciton and free-carrier populations and contradicts both established semiconductor theory and experimental observations. Saha-like relations can be used to describe the phase diagram between free excitons and free charges only if they account explicitly for many-body corrections. In any case, the Mott model remains the best-established and easiest to use method that should always be preferred when describing photoexcited semiconductors. Clarifying these distinctions is also essential for a reliable modeling of semiconductor devices in operando.
